# American Dental Colleges

**Published:** 1895-09

**Authors:** 


### Editorial, Etc.

### AMERICAN DENTAL COLLEGES.

We have elsewhere noticed *The Transactions of the American Dental Association*, but we may here perhaps be allowed to consider at greater length the report which was made in Section II., which dealt with Dental Education, Literature and Nomenclature. From this it appears that the National Association of Dental Faculties still continues to exercise an influence for good. Perhaps no more important or hopeful movement has marked the progress of the Profession in recent years, than the voluntary banding together of the best American Schools. Instead of students drifting to other centres where a shorter curriculum was demanded, it has been found that this class of the community was wise in its generation, and really helped and supported the new movement. There was a decrease in the number of graduates in 1893 and 1894 due to the operation of the three years course, but a similar condition of things is noticed with us whenever the curriculum is extended, and it is a matter which generally rights itself before long. At the present time there are thirty-two teaching Institutions which belong to the National Association; those not on the roll are chiefly the ones recently organized, and even these, with a view of rendering themselves eligible for membership, conduct themselves with that end in view. One of the great drawbacks the Association of Faculties seem subject to is the ease with which new Dental Schools can spring into existence. Each State is able to do as it pleases, and during the last ten years the number of schools has doubled. The opinion is expressed that in the future the increase is likely to become even greater, in consequence of the existence of what is described as a "fad" on the part of medical schools to establish a dental department. Out of the forty-four dental colleges, twenty-seven are "operated" in conjunction with medical departments. The criticism offered upon this easy method of increase (some of the medical schools requiring no additional charter and already having teaching provision in several of the subjects) is to the effect that it may be profitable to the medical school but is not calculated to redound to the credit of the dental profession. We are, however, glad to read the first portion of the following quotation, and at the same time can appreciate the humorous allusion in the latter part: "It is not the desire of the Section to deprecate such a condition, for many of the schools thus connected are among the best of the country; but it should be borne in mind that there are more than two hundred and fifty medical colleges in the United States, and the addition of a vermiform appendix to each of these may fasten upon the dental profession a dangerous disease." We do not altogether agree that a dental department should be compared with that *cul-de sac* of the human economy, the function of which appears doubtful unless it be to serve as an instance of a useless and sometimes dangerous survival.

Another matter alluded to is the necessity which exists for some more or less uniform test as to a student's proficiency in general education before entering upon the professional curriculum. At present every school judges its own candidates, and this leads to "the admission to the ranks of the dental profession to many whose fitness may be questioned with propriety." It is suggested that the degree of Bachelor of Arts, or its equivalent, should be demanded, but then the stipulation is necessary "provided that it is obtained from a reputable university." Another important thing recommended is the extension of the school year to nine months, and the increase of curriculum to four years. Owing to vacation, and other interruptions, it is possible under the existing rules for a man to complete his education without having *actually* devoted more than twelve months to the study of dentistry.

There are many other subjects worthy of consideration in the report before us which we hope to find an opportunity of placing before our readers. The earnest men who are in the fore-front of the battle of professional progress in America will doubtless continue their efforts. Too little is known in other countries (where circumstances are so different) of the difficulties of the struggle, but our Trans-Atlantic brethren may rest assured that they have the sympathy of British practitioners, who (in spite of occasional misunderstandings) welcome good work for the advancement of the profession wherever it may be done. We may perhaps just notice that in the discussion which followed the reading of the report, Dr. Abbott referred to the existence of a "Diploma Mill" in Kansas City, legally chartered, and carrying on business in selling diplomas; Dr. Ottofy was also of the opinion that there might be others which escaped observation. These remarks may be of interest to our correspondent who signs himself "Registered."—*Editorial in British Dental Journal.*

